# Growth rate of ovulatory follicles during the first ovulatory oestrus (after seasonal anoestrus) and subsequent oestrous period in Irish Draught mares

**DOI:** 10.1186/2046-0481-66-4

**Published:** 2013-03-12

**Authors:** John R Newcombe, Juan Cuervo-Arango

**Affiliations:** 1 Equine Fertility Clinic, Warren House Farm, Brownhills WS8 6LS, UK; 2 Departamento de. Medicina y Cirugía Animal, Facultad de Veterinaria, Universidad CEU Cardenal Herrera, Moncada, Spain

**Keywords:** Irish Draught, Mare, Follicle, Growth rate, First ovulatory oestrus

## Abstract

It is believed that during the spring transition, the developing follicle tends to grow more slowly, persist longer and grow to a larger diameter prior to ovulation than at subsequent oestrus periods. A general suspicion, that the first ovulation of the year is less fertile than subsequent ovulations could be explained by a slower growth rate of the ovulatory follicle during transition with the consequent production of a subfertile oocyte. By detailed serial examination of the same group of Irish Draught mares over three winter/spring periods, no significant difference was found in either growth rate or pre-ovulatory diameter when compared with subsequent ovulations. Mean growth rates over the ten days prior to ovulation were 2.20 mm/day (range 1.18 to 3.64) and 2.19 mm/day (range 1.25 to 3.41) for first and subsequent ovulations respectively. Mean maximum pre-ovulatory diameters were 44.7 mm (range 35 to 59) and 43.5 mm (range 31 to 57.5) for first and subsequent ovulations respectively. The impression gained by practitioners that the first follicle develops more slowly during the transition to the first ovulation of the season may be due to less frequent examinations and consequently a failure to observe and record that follicles may grow and then regress during this period. The largest follicle observed a few days previously is not necessarily the same large follicle found at a later examination.

## Background

Practitioners tend to believe that during the spring transitional period, large developing follicles persist for longer periods prior to ovulation than during subsequent oestrous periods. This view is supported by a study of Ginther [1] who reported that prior to the first ovulation of the year, follicles reached 20 mm earlier and grew more slowly (2.6 v. 3.6 mm/day) than after the end of the first cycle. In addition it was found that the ultimate preovulatory diameter was larger 51 v. 44 mm [1]. A general suspicion, backed by some evidence (Cuervo-Arango and Clark 2009), that the first ovulation of the year is less fertile than subsequent ovulations could be explained by a slower growth rate of the ovulatory follicle during transition with the consequent production of a subfertile oocyte. The average follicle is said to develop at a rate of 2.7 mm/day [2] but this may vary from 2 to 5 mm/day or even no growth over the 3–4 days preceding ovulation [3]. Frequently, when observations are made at intervals of less than 24 h in the immediate pre-ovulatory period, the largest recording is not the one made just before ovulation [4]. The apparent reduction in diameter in this period may be due to a problem of measurement of an irregular shaped softening follicle or to actual leakage of follicular fluid at the beginning of ovulation. Ginther [5] found that ovulation occurred 4.2 days after the dominant follicle attained a diameter of 35 mm, a growth rate of 2.4 mm/day for a pre-ovulatory diameter of 45 mm.

The aim of this retrospective study was to compare any differences in growth rate and pre-ovulatory follicle diameter between first and subsequent cycles in the same mares.

## Methods

During the autumn/winter/spring periods of three seasons, 32 pure and crossbred Irish Draught mares were stabled and maintained under extended daylength of 16 hours light beginning December 16^th.^ Prior to the first ovulatory oestrus following a period of acyclicity (seasonal anoestrus), all mares were examined at intervals of no more than 7–10 days, more frequently when follicular activity increased and at least once daily for at least 11 days before ovulation and when a follicle reached 35 mm. All follicles of 20 mm or larger were measured and recorded. In the absence of any follicle in either ovary larger than 20 mm, the diameter of any smaller follicle was also recorded. It was therefore possible to trace back from the time of ovulation, the diameter of the ovulatory follicle during its development. Where over several days, one follicle in an ovary regressed, it was assumed that another initially smaller follicle in the same ovary had grown to overtake the larger and progressed to ovulation. All ovulations were spontaneous and not induced with hCG or GnRH. Day 0 was the day preceding ovulation.

Follicles were measured by freezing what was considered to be the most representative image and the follicle diameter measured with electronic callipers from the mean of two right angle diameters. However large follicles close to ovulation were frequently of irregular shape which resulted in some variation in sequential diameters. This was due to the difficulty in always maintaining a truly representative image in two dimensions, of an irregular 3-dimensional body. In most cases, more than one observation was made during the 24 hour period before ovulation. The final diameter used for the analysis was that of the largest diameter recorded in that 24 hour period. Not infrequently an even larger diameter had been recorded in the period 24–48 h before ovulation.

Mean follicle diameters were calculated. All data were tested for normality (Anderson-Darling Test). Data not normally distributed were ranked. Ovulation data from different months were pooled into two groups (first or second half of the ovulatory season) for first and subsequent ovulations. The differences in pre-ovulatory follicle diameter and mean daily growth rate between the first and subsequent ovulations of the year for the first and second halves of the observation period was analysed using paired *t*-test. The degree of repeatability in follicle diameters and growth rate was tested using Pearson correlation.

## Results

Dates of the last ovulation prior to anoestrus varied from October to December. Four mares cycled continuously through the winter and into the next spring thus providing cyclic data in January and February. First ovulation dates ranged from January 14^th^ to May 22^nd^ and from January 15^th^ to June 16^th^ for subsequent cycle ovulations respectively. Some mares had periods of non-ovulatory follicular activity with endometrial oedema but were not teased for oestrous behaviour. Other mares ovulated directly from anoestrus with no transitional phase.

Each group was sub-divided in halves chronologically to test the effect of season. The ovulation dates in the first half of each group ranged from January 14^th^ to February 22^nd^ (first ovulations) and from January 15^th^ to March 12^th^ (subsequent ovulations), and in the second half groups from February 25^th^ to May 22^nd^ (first ovulations) and from March 14^th^ to June 16^th^ (subsequent ovulations).

Thirty two ovulatory follicles were followed in 32 individual mares prior to their first ovulation of the season. Another 34 ovulatory follicles were recorded at subsequent cycles in the same mares, at 28 second and 6 third cycles. The intensity of examinations in this group was less than during the first oestrus. Consequently although the number of observations was 34 for the two days before ovulation this reduced as the interval to ovulation increased. By Day −7, a time when not all mares had undergone luteolysis, the number of observations was only 14 of the 34, making mean values from Day −7 to Day −11 less reliable.

**Figure 1:**
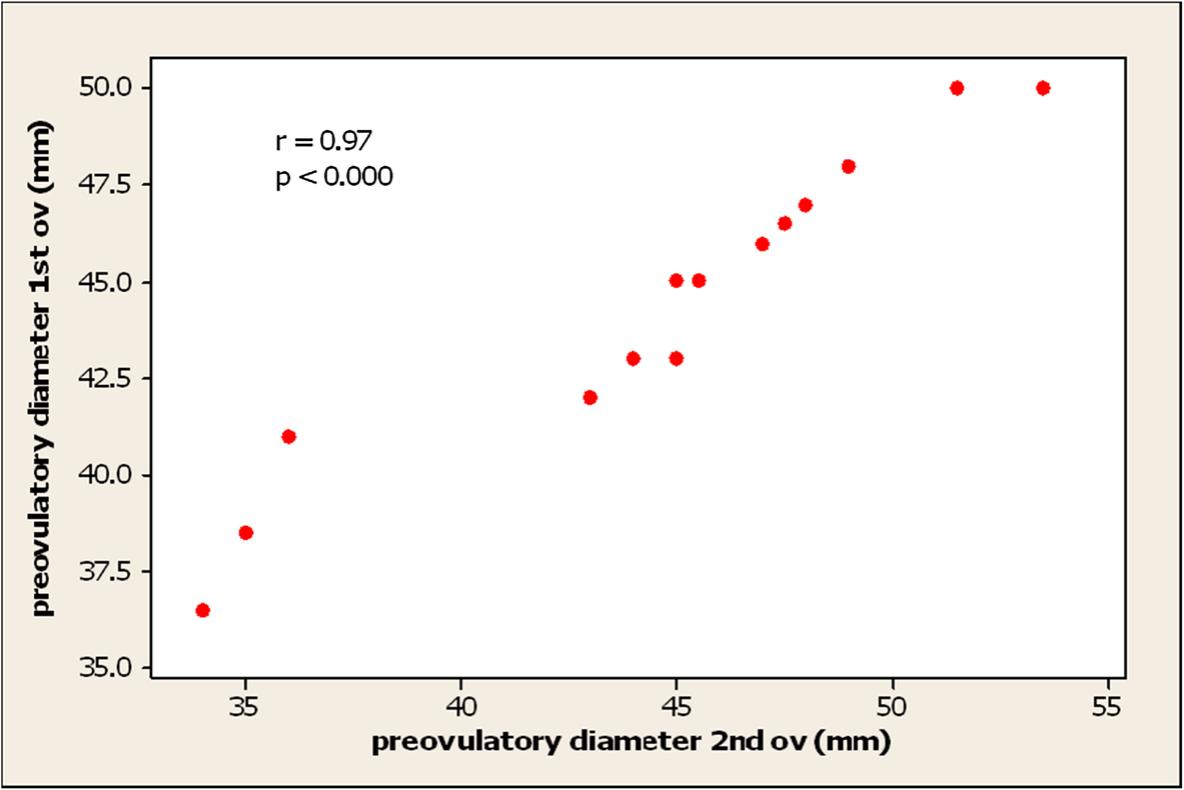
**Scatter plot correlating pre-ovulatory follicular diameter in individual mares.** (1^st^ ovulations with 2^nd^/3^rd^ ovulations. First halves only).

There was no statistical difference between groups (Table 1) (p > 0.05). The degree of repeatability in follicular diameters in consecutive cycles was highly significant for both halves of the season (p < 0.000), r = 0.97 and r = 0.98 for first and second halves. This is illustrated by a scatter plot for first halves (Figure 1). Mean maximum follicle diameters in the period 24 hours before ovulation were 44.7 mm and 43.5 mm for first and subsequent ovulations respectively, with a range of ovulatory diameters from 35 to 59 mm and from 31 to 57.5 mm respectively. There was no statistical differences in mean diameter between groups viz. 1.2, 0.1, 0.2, 0.5, 0.6, 0.3, 1.9, 0.8, 0.5, 0.7, -1.4 and −3.1 mm for days 0 to −11 respectively (Table 1).

**Table 1:** Pre-ovulatory follicular growth rates: a comparison between the first ovulation after winter anoestrus and later ovulations in the same mares

	**Follicle diameters up to 11 days before ovulation (mm)**											
Days before ovulation	−11	−10	−9	−8	−7	−6	−5	−4	−3	−2	−1	0
**First ovulations of the season**												
**Jan 14th to Feb 22nd**												
No. of observations	15	16	16	16	16	16	16	16	16	16	16	16
Mean diameter	20	23.5	26	27.7	30.1	33.1	34.3	36.2	39.1	41.7	43.5	44.6
**Feb 25th to May 22nd**												
No. of observations	16	16	16	16	16	16	16	16	16	16	16	16
Mean diameter (mm)	20.3	22.3	25	27.5	30.1	33.3	35	38	40.2	42.1	42.7	44.8
**Jan 14th to May 22nd**												
Total no. of observations	31	32	32	32	32	32	32	32	32	32	32	32
**Mean diameter (mm)**	**20.1**	**22.9**	**25.5**	**27.6**	**30.1**	**33.2**	**34.6**	**37.1**	**39.6**	**41.9**	**43.1**	**44.7**
Range (mm)	<10-36	<10-37	10-41	10-43	10-44	12-45	15-48	18-48	20-53	25-55	31-57	35-59
**Subsequent ovulations**												
**Jan 15th to Mar 12th**												
No of observations	2	4	3	4	9	5	11	11	14	15	14	17
Mean diameter (mm)	19	24.8	20.3	25.8	28.2	32.6	34.7	36.6	39.3	41.9	44.8	44.2
**Mar 14th to June 16th**												
No. of observation	3	3	3	5	5	8	7	11	13	14	17	17
Mean diameter (mm)	26	23.6	29.2	28.2	31.4	30.4	33.7	36.5	39	41.5	41.6	42.8
**Jan 15th to June 16th**												
No. of observations	5	7	6	9	14	13	18	22	27	29	31	34
**Mean diameter (mm)**	**23.2**	**24.3**	**24.8**	**27.1**	**29.3**	**31.3**	**34.3**	**36.5**	**39.1**	**41.7**	**43**	**43.5**
Range (mm)	14 - 32	17 - 34	18 -36	16 -37	15 -41	23- 39	24 - 43	24.5-47	26 - 52	27.5-52	31.5-55	31-57.5
Difference in mean diameters	−3.1	−1.4	0.7	0.5	0.8	1.9	0.3	0.6	0.5	0.2	0.1	1.2
Mean growth rate (mm/day)	Day −11 to 0		Day −8 to 0		Day −5 to 0							
First ovulations	2.20		2.14		2.02							
Subsequent ovulations	2.19		2.37		1.84							
Difference	0.01		0.23		0.18							

Mean growth rates for first and subsequent ovulations were not different, 2.20 and 2.19 mm/day from Day −11 to ovulation (p > 0.05), 2.14 and 2.37 mm/day from Day −8 to ovulation (p > 0.05), and 2.02 and 1.84 mm/day from Day −5 to ovulation (p > 0.05). Growth rate of individual follicles varied in the extreme within both groups and was not affected by time of year. The degree of repeatability in mean daily growth rate during the follicular phase before the first ovulation of the year and subsequent ovulations was poor (r = 0.1, p > 0.05) for both halves of the season.

There was no evidence that follicles to grew more rapidly as the season progressed. Table 2 shows that follicle growth rates in individual mares ranged from 1.18 to 3.64 mm/day and 1.25 to 3.41 mm/day for first and subsequent ovulations respectively from Day −11 to Day 0. Individual growth rates between Day −8 and Day 0 ranged from 0.88 to 3.73 mm/day and from 0.88 to 3.88 mm/day respectively.

**Table 2:** Individual mare variation in follicle growth rates and ultimate preovulatory diameter

	**Ultimate diameter (mm)**	**Growth rate from Day −11 (mm/day)**	**Growth rate from Day −8 (mm/day)**		**Ultimate diameter (mm)**	**Growth rate from Day −11 (mm/day)**	**Growth rate from Day −8 (mm/day)**
**First ovulations**				**2nd/3rd ovulations**			
**First half**				**First half**			
1	50	3.64	3.75	1	53.5	2.95	2.56
2	50	2.95	3.38	2	51.5	3.41	2.23
3	48	2.64	1.63	3	49	2	1.5
4	47	1.95	1.5	4	48	3	2.45
5	46.5	2.23	2.06	5	47.5	No data	3.19
6	46	1.73	1.31	6	47	No data	2
7	46	1.77	1.81	7	47	No data	3.88
8	46	2.36	2.06	8	47	No data	3.38
9	45	2.05	2.19	9	45.5	No data	3.19
10	45	1.73	1.31	10	45	1.91	2.79
11	43	2.18	2.5	11	45	No data	1.93
12	43	2.09	2.13	12	44	No data	1.19
13	42	1.82	2.75	13	43	1.82	1.14
14	41	1.82	1.88	14	36	1.91	3
15	38.5	2.41	2.31	15	35	2.09	1.89
16	36.5	1.29	1.31	16	34	No data	1.86
				17	33	No data	1.57
**Mean**		**2.17**	**2.12**			**2.39**	**2.34**
**Second half**							
1	59	3.55	3	1	57.5	No data	3.87
2	54.5	3.05	3.06	2	51	2.09	2.3
3	50	1.18	0.88	3	46.5	No data	3.58
4	48.5	2.32	2.06	4	46.5	No data	3.25
5	48	2.56	2.75	5	46	No data	3.08
6	48	2.36	2.13	6	45.5	No data	2.58
7	46.5	2.09	1.94	7	44.5	No data	1.56
8	45	1.36	1.19	8	44	No data	1.36
9	43	1.68	1.75	9	43.5	2.32	3.25
10	42.5	2.5	2.13	10	43	No data	2.29
11	41.5	2.05	2.5	11	41.5	No data	3.5
12	41	2.82	2.63	12	41	No data	1.5
13	39.5	2.05	2.19	13	40.5	No data	2.92
14	39	2.09	1.75	14	38	2.36	0.88
15	36	2.36	3.25	15	35.5	No data	2.5
16	35	1.64	1.38	16	32.5	1.25	1.19
				17	31	1.4	1.38
**Mean**		**2.23**	**2.16**			**1.88**	**2.41**
Both halves							
**Mean**		**2.2**	**2.14**			**2.19**	**2.37**

Follicular growth was apparently zero or negative in the three days before ovulation in 10 (31%) and 7 (21%) individual mares in the first ovulation and subsequent ovulation groups respectively. Conversely, growth rates of 5 to 9 mm in the last two days occurred in 8 and 7 mares in the two groups.

## Discussion

The growth rate of follicles varied widely between individual mares as did the pre-ovulatory diameter. However the correlation in pre-ovulatory diameter between consecutive cycles was very strong as has been shown before [6,7]. In studying growth rates in the same group of mares by serial daily examination and diligent recording of all follicles, there was no evidence found that following a period of winter anoestrus, the mean growth rate of the ovulatory follicle was any slower than follicles which begin development during the dioestrous phase of a normal cycle. The mean growth rate was in fact marginally slower in cyclic mares when measured over the 12 days before ovulation. However a comparison of the early part of follicular development which occurs during dioestrus with that during the transition phase was not justified. The observation that some mares in both groups had zero or negative growth between Day −2 and Day 0 was possibly an artefact caused by measurement difficulties already discussed.

This report differs fundamentally from that of Ginther [1] who found both a slower rate of growth and a larger pre-ovulatory follicle diameter in transitional pony mares than at the second ovulation. However that study was based upon only 16 and 9 ovulations in a different breed. In the current study it would not be difficult to identify at least a number of individual mares whose follicle development would support Ginther’s findings. The growth rates varied widely between individuals, from 0.88 and 0.88 mm/day to 3.75 and 3.88 mm/day in both groups while pre-ovulatory diameters varied from 35 and 31 mm to 59 and 57.5 mm. Thus both the means and ranges of growth rates, and pre-ovulatory diameters were almost identical for both groups. Previous studies found non-significant differences in pregnancy rate between ovulations from first and subsequent cycles in thoroughbred mares, 65.2% v. 76.0% [8] and 67.2% v.73.7% (Newcombe and Cuervo-Arango: unpublished). In the latter study, the multiple pregnancy rate was 50% higher at second cycles (23.8% v. 15.7%). This evidence, that the first ovulation of the year is less fertile, cannot be due to unduly prolonged development or persistence of the ovulatory follicle.

## Conclusions

The impression gained by practitioners that the first follicle develops more slowly during the transition to the first ovulation of the season may be due to less frequent examinations and consequently a failure to observe and record all follicles that may grow and then regress during the transitional period. The largest follicle observed a few days previously is not necessarily the same large follicle found at a later examination.

## Competing interest

The authors declare that they have no competing interests.

## Authors’ contributions

JRN collected the data, designed the experiment and wrote the manuscript. JCA performed the statistic analyses and revised the manuscript. Both authors read and approved the final manuscript.
